# Complete mitochondrial genome sequence of *Tapinoma melanocephalum* (Hymenoptera: Formicidae)

**DOI:** 10.1080/23802359.2019.1674205

**Published:** 2019-10-09

**Authors:** Yimin Du, Xiang Song, Haizhong Yu, Zhanjun Lu

**Affiliations:** a School of Life Sciences, Gannan Normal University, Ganzhou, Jiangxi, China;; b National Navel Orange Engineering and Technology Research Center, Ganzhou, Jiangxi, China

**Keywords:** Formicidae, mitochondrial genome, *Tapinoma melanocephalum*, phylogenetic analysis

## Abstract

*Tapinoma melanocephalum* is a ubiquitous invasive species and widely distributed in subtropical and tropical regions around the world. Here, we sequenced and annotated the complete mitochondrial genome (mitogenome) of *T. melanocephalum*. This mitogenome was 15,499 bp long and encoded 13 protein-coding genes (PCGs), 22 transfer RNA genes (tRNAs), and 2 ribosomal RNA unit genes (rRNAs). Compared to other Formicidae species, gene order of *T. melanocephalum* was not conserved and one tRNA cluster *trnW*-*trnC*-*trnY* converted to *trnW*-*trnY*-*trnC*. The whole mitogenome exhibited heavy AT nucleotide bias (79.5%). All PCGs started with the standard ATN codons. Except for *cox1* and *nad5* end with the incomplete codon T−, all PCGs terminated with the stop codon TAA. Phylogenetic analysis showed that *T. melanocephalum* got together with three same subfamily Dolichoderinae species and one Dorylinae species, indicating the close relationship of Dolichoderinae and Dorylinae.

*Tapinoma melanocephalum* (Fabricius), the ghost ant, is a common invasive and widely distributed ant species which has spread across the subtropical and tropical regions around the world, and even invades temperate regions as a result of global commerce (Choe et al. 2009; Zheng et al. 2018). This species often nest in rotten wood, soil, walls of houses, dry grass clumps, and plant stems. The colonies of *T. melanocephalum* are polygynous and may bud into several satellite sub-colonies making them difficult to control (Luo and Chang 2013).

Specimens of *T. melanocephalum* were collected from Ganzhou City, Jiangxi Province, China (25°47′N, 114°51′E, April 2019) and were stored in Entomological Museum of Gannan Normal University (Accession number GNU-TM052). After morphological identification, total genomic DNA was extracted from tissues using DNeasy DNA Extraction kit (Qiagen, Hilden, Germany). The mitogenome sequence of *T. melanocephalum* was generated using Illumina HiSeq 2500 Sequencing System (Illumina, San Diego, CA). In total, 6.4 G raw reads were obtained, quality-trimmed, and assembled using MITObim v 1.7 (Hahn et al. 2013). By comparison with the homologous sequences of other Formicidae species from GenBank, the mitogenome of *T. melanocephalum* was annotated using software GENEIOUS R8 (Biomatters Ltd., Auckland, New Zealand).

The complete mitogenome of *T. melanocephalum* is 15,499 bp in length (GenBank accession no. MN397938), containing the typical set of 13 protein-coding genes (PCG), 2 rRNA, and 22 tRNA genes, and one non-coding AT-rich region. Except for the tRNA cluster *trnW*-*trnC*-*trnY*, which converted to *trnW*-*trnY*-*trnC*, all other gene orders were conserved and identical to most other previously sequenced Formicidae (Williams and Wernegreen 2013; Kim et al. 2016; Yang et al. 2016; Lee et al. 2018). The overall base composition of the mitogenome was estimated to be as follows: A, 40.3%, T, 39.2%, C, 14.0%, and G, 6.5%, with a high A + T content of 79.5%. Four PCGs (*nad4*, *nad4l*, *nad5*, and *nad1*) were encoded by the minority strand (N-strand) while the other nine were located on the majority strand (J-strand). All PCGs started with the standard ATN codons (five ATG, four ATT, three ATA, and one ATC). Except for *cox1* and *nad5* end with the incomplete stop codon T−, all other PCGs terminated with the stop codon TAA. The 22 tRNA genes vary from 61 bp (*trnS1*) to 72 bp (*trnA*). The lengths of *rrnL* and *rrnS* in *T. melanocephalum* were 1346 and 742 bp, with the AT contents of 84.5 and 86.8%, respectively.

All 13 mitochondrial PCG sequences were extracted from the mitochondrial DNA sequences of 19 closely related taxa of Formicidae, including one outgroup species from Vespidae. The phylogenetic tree was constructed using the maximum-likelihood method through raxmlGUI 1.5 (Silvestro and Michalak 2012). Results showed that the newly sequenced species *T. melanocephalum* got together with three same subfamily Dolichoderinae species (*Linepithema humile*, *Dolichoderus sibiricus*, and *Leptomyrmex pallens*) and one Dorylinae species (*Ooceraea biroi*) (Figure 1), indicating the close relationship of Dolichoderinae and Dorylinae. In conclusion, the complete mitochondrial genome sequence of *T. melanocephalum* provides an important molecular framework for further phylogenetic analyses of Formicidae.

**Figure 1.**
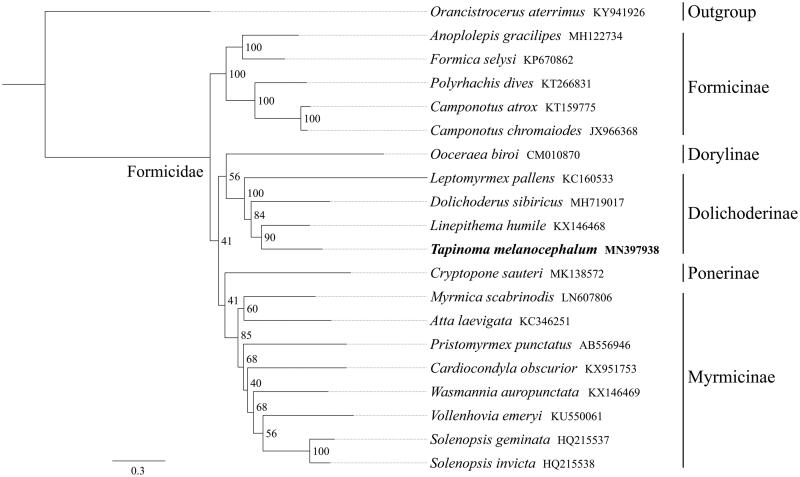
Phylogenetic relationships based on the 13 mitochondrial protein-coding genes sequences inferred from RaxML. Numbers on branches are Bootstrap support values (BS).
